# Vehicle Based Laser Range Finding in Crops

**DOI:** 10.3390/s90503679

**Published:** 2009-05-15

**Authors:** Detlef Ehlert, Rolf Adamek, Hans-Juergen Horn

**Affiliations:** Leibniz-Institute for Agricultural Engineering Potsdam-Bornim, Max-Eyth-Allee 100, 14469 Potsdam, Germany; E-Mails: radamek@atb-potsdam.de; jhorn@atb-potsdam.de

**Keywords:** Precision Agriculture, Crop sensors, Laser rangefinder

## Abstract

Laser rangefinders and laser scanners are widely used for industrial purposes and for remote sensing. In agriculture information about crop parameters like volume, height, and density can support the optimisation of production processes. In scientific papers the measurement of these parameters by low cost laser rangefinders with one echo has been presented for short ranges. Because the cross section area of the beam increases with the measuring range, it can be expected that laser rangefinders will have a reduced measuring accuracy in small sized crops and when measuring far distances. These problems are caused by target areas smaller than the beam and by the beam striking the edges of crop objects. Lab tests under defined conditions and a real field test were performed to assess the measuring properties under such difficult conditions of a chosen low cost sensor. Based on lab tests it was shown that the accuracy was reduced, but the successful use of the sensor under field conditions demonstrated the potential to meet the demands for agricultural applications, Insights resulting from investigations made in the paper contribute to facilitating the choice or the development of laser rangefinder sensors for vehicle based measurement of crop parameters for optimisation of production processes.

## 1. Introduction

In the future agriculture will have to be both competitive and environmentally friendly. This aim can be achieved by reducing the consumption of natural resources and increasing the input of information into the production process [1]. In agricultural production, crop height, degree of coverage, and biomass density are all important parameters for the assessment of crop plants. Based on these parameters, expected crop yields can be appraised and the amount of fertilisers and pesticides for the site-specific crop management can be optimised. Furthermore, in combine harvesting parameters such as ground speed or the rotation speed of functional units (rasp-bar cylinder, cutter head) can be adapted specifically to on-site crop conditions [2].

Laser rangefinders currently available on the market use varied measuring principles: light time-of-flight, phase modulation, interferometry, and triangulation. In many cases the first three principles are combined into the technique known as time-of-flight measurement. Triangulation sensors measure short ranges (maximum a few meters) with high accuracy, while time-of-flight sensors are suitable for both short and far ranges. Today's commercially available laser rangefinders differ greatly in price and performance parameters. Simple laser rangefinders can cost less than 1,000 € while high end laser scanner systems can cost up to about one million Euros.

There are spaceborne laser scanner systems for detecting wide swaths from satellites and airborne scanner systems (ALS) for detecting medium ranged areas from aircraft (500-1,000 m) and helicopters (200-300 m) [3,4]. These are very costly technologies (>500,000 €), used mainly for remote sensing of large scale landscapes [5-8], urban areas [9], and in forestry [10,11]. Terrestrial laser scanner systems (TLS) are e.g. suitable for surveying purposes, cultural heritage, city modelling or architectural applications [12], for mobile road mapping system [13] and for the determination of forest inventory parameters [14,15]. The prices for these systems can be 50,000 € and more. Buildings, the surface of landscapes and also trees have normally large dimensions and show marginal changes over long periods, but agricultural crops have high growth dynamics and a short life cycle on the order of months, so in order to manage agricultural crop production, current information is needed - sometimes within hours or seconds. Therefore, many agricultural vehicles should be equipped with their own low cost sensor to perform real-time operations, and the above described sensor systems are unacceptable for characteristic agricultural applications.

Idealised, the light intensity over the cross section area of a laser spot can be characterised as a three dimensional normal (Gauss) beam. The beam emitted from the sensor has a device specific cross section area and depends on the measuring range [16]. Baltsavias presented basic relations and formulas for treating lasers, laser ranging, and airborne laser scanners [17]. Wagner *et al.* [18] introduced the Gaussian decomposition and calibration of a novel small-footprint full-waveform digitising airborne laser scanner. From the theoretical aspects it follows that the measuring properties of laser rangefinders depend on beam parameters, hard- and software configuration of measuring system, measuring range and reflection properties of target objects. Because the measuring properties are sensor specifically they must be investigated for the intended application.

In contrast to industrial applications which almost always take place under in-door conditions, the tasks under real field conditions are more difficult. Besides impacts from vibrations and dust, further problems result from out-door operation under very different weather and illumination conditions. In order to measure during intensive sunlight, sensors are supplied from the manufacturer with a 3B classification. A Class 3B laser is hazardous if the eye is exposed directly, but diffuse reflections such as from paper or other matte surfaces are not harmful. This can lead to labour safety problems. Furthermore, in crop production it is necessary to measure crop target objects (leafs, stems, soil) with very different shapes, inclinations and relative small dimensions while the vehicle is in movement. Characteristic examples for these conditions are measurements in grass and cereals, which have such a structure. The footprints of the beams used in airborne and terrestrial remote sensing of trees are in the range of dm and more. These scanners are very expensive and can discriminate multiple echoes. Currently sophisticated methods of airborne laser scanning are in development. Analysing in more detail the reflection signals (full waveform) can improve the information that is gathered about the target objects [19-21].

Low cost sensors do not have this option. Under such conditions the problem of how to assess the readings of simple sensors with only one echo when the beam targets different crops or soil surfaces remains unanswered.

As far as the beam guidance aspect is concerned, we can distinguish between sensors with a fixed beam and those with a scanning beam. For laser scanners there are limited opportunities for the users to influence the movement of the beam. For lasers with a fixed beam individual solutions can be developed by moving the entire sensor housing with some corresponding kinematics.

In the field of agricultural engineering research, low cost laser rangefinders available in the marketplace have been investigated in both horticulture and in agriculture applications. In horticulture Tumbo *et al.* measured the canopy volume and structure in citrus [22]. Walklate *et al.* compared different spray volume deposition models using LIDAR measurements of apple orchards [23]. Sanz *et al.* reported on advances in the measurement of structural characteristics of plants (peach trees) with a LIDAR scanner [24]. Escola *et al.* investigated a variable dose rate sprayer prototype for tree crops based on sensor measured characteristics [25].

In agriculture Thösink *et al.* made a first test to measure the height of oat plants [26]. To calculate the crop height, the level of soil surface was discriminated from the distribution of height classes. Kirk *et al.* estimated in a comparative study the canopy structure from laser range measurements and computer vision [27]. Ehlert *et al.* measured crop biomass density in oilseed rape, winter rye, winter wheat and grassland by laser triangulation [28]. Lenaerts *et al.* predicted crop plant density using LIDAR-sensors [29].

Ehlert *et al.* assessed low cost laser rangefinders for vehicle-based measurement of crop biomass [2]. High functional correlations were found between mean reflection height h_Rmean_ (m) – calculated from measured reflection range and sensor height – and fresh crop biomass density FMD (kgm^-2^). In oilseed rape, winter rye and winter wheat crops, the goodness of fit for a linear regression was more than 0.90 (R^2^ > 0.9). In grassland (pasture) the accuracy was lower. This can be explained by the occurrence of several plant species with variable morphology and the small dimensions of leaves and stems (Table 1).

**Figure 1.**
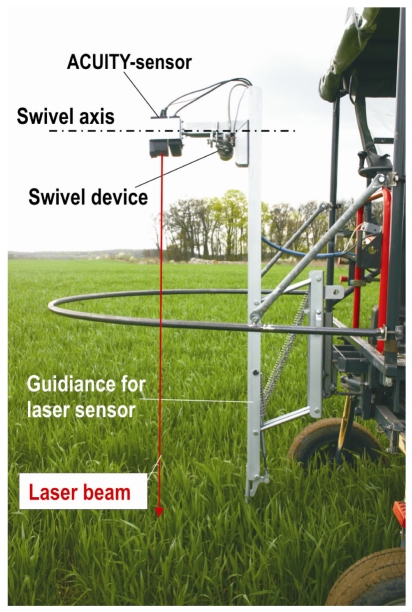
Arrangement of laser rangefinder and swivel device (horizontal axis) on a basic vehicle for measuring crop parameters.

In these measurements the beam was either directed down at the crop plants or was pivoted around a horizontal axis of ±15° (Figure 1). Due to the mounting height of the sensor the measuring range was less than 2.5 m. Under these conditions the diameter of laser beam was in the range of millimetres. The arrangement of measuring points followed either a straight line or a sinusoidal line with amplitude less than 1.34 m.

Lenaerts *et al.* tested two LIDAR-Sensors for predicting crop stand density under lab conditions [29]. The sensors were mounted in 2.85 m height on a combine harvester. In this paper it was concluded that a sufficient measuring distance und a small beam diameter are necessary to prevent measurement failures caused by spots larger than the target objects and by beams striking the edges of objects.

Agricultural equipment for application of fertilisers and plant protection agents normally have working ranges of more than 20 m. High end combine harvesters can today achieve cutting widths in the range of 10 m. To acquire crop plant data in a representative manner for these working widths in front of the machines the measurements have to acquire a broader strip and from these higher ranges result the problems described above. From these working conditions follows that laser rangefinders should be able to survey crop parameters up to a distance of about 15 m. For this measuring range no experience in agricultural crops is available.

Therefore, in this paper the following parameters were investigated for a chosen laser rangefinder:

- Variation of range readings depending on measuring distance and reflection medium under static conditions
- Distribution of the light intensity inside the spot cross section
- Measuring properties for multiple reflection levels inside of the beam
- Measuring properties for variable velocities of target medium and measuring distances
- Measurements under same conditions in a real crop

## 2. Material and Methods

For the investigations an ACUITY AccuRange 4000-LIR sensor (Schmitt Measurement Systems, Inc., USA) was used. The sensor was chosen because in preceding tests it provided the best results [2]. It has a co-axial beam working with near infrared laser light according to the phase modulation principle (Table 2). The sensor generates only one echo for range measurements.

### 2.1. Variation of range readings depending on measuring distance and reflection medium under static conditions

To investigate the repeatability of measurements (random error) under static conditions, the variance and the coefficient of variation of the readings were estimated for the short, medium and far measuring ranges. For this purpose the sensor was fixed on a tripod with a horizontal beam orientation. The reflection media were a white sheet of paper, plant leaf and soil (sand). The reference distance was taken in a first step from a tape to adjust the short, medium and far ranges. The systematic error (offset) was not estimated because under real measuring conditions in crops, the height of reflection points is calculated for crop assessment [2]. For this the measuring system is configured in such a way that the ground level reflection height is zero. In this case constant offsets would be eliminated. Differences in sensor height related to the basic vehicle are avoided by the height guidance unit, according to Figure 1.

### 2.2. Distribution of light intensity inside the spot cross section

To investigate the light intensity distribution inside of the spot the sensor was fixed on a tripod with a horizontal axis. A white sheet of paper was placed perpendicular to the beam at three distances. Because laser light with a wavelength of 780 nm and cannot be observed identified by the human eye a digital camera and a ruler were used to indicate the intensity distribution inside the laser beam.

### 2.3. Measuring properties for multiple reflection levels inside of the beam

**Figure 2.**
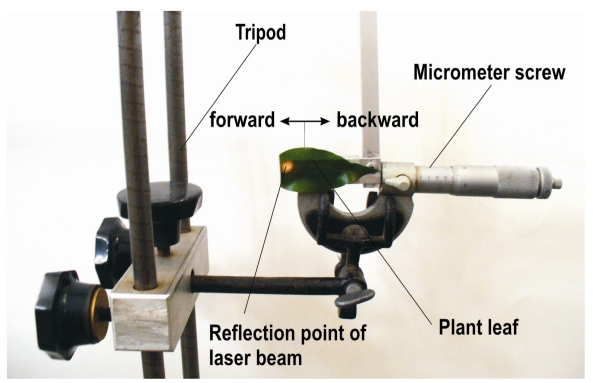
Investigation of sensor behaviour for two reflection levels (plant leaf and background).

Laser beams have a certain diameter. Because of this feature a beam can produce two or more reflexion levels when targeted on fine structured objects and therefore failure readings can be generated. Hence, the sensitivity inside the beam was investigated. For this purpose the horizontal oriented beam of the sensor investigated was directed on a rear medium distance surface of sandy soil (reflection level B). Then a second reflection surface consisting of a leaf from a ficus plant (reflection level A) was installed in front (Figure 2). The leaf had a straight edge cut with a shear and was fixed in the movable part of a micrometer screw. The fixed part of the micrometer screw was clamped on a tripod. With this arrangement it was possible to move the edge of the leaf forwards and backwards through the laser beam in a stepwise fashion with high accuracy. Because the distances to both reflection surfaces were known, conclusions about the measuring properties were possible.

### 2.4. Measuring properties for variable velocities of target medium and measuring distances

**Figure 3.**
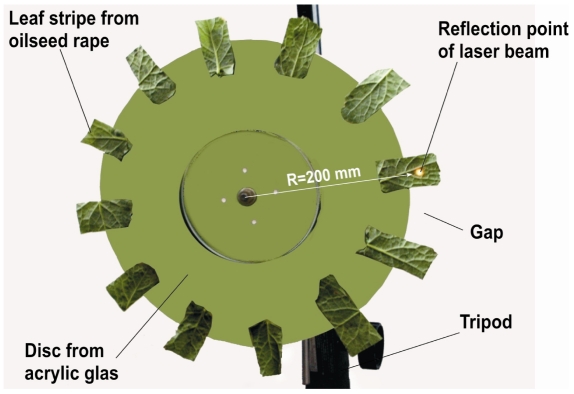
Rotation disc to investigate the sensor measuring properties for two reflection levels (teeth from oilseed rape and background) under dynamic conditions.

To investigate how different measuring ranges and target velocities influence the measurement properties, a disc (diameter 380 mm) made from acrylic glass was used (Figure 3). The disc was covered up with green paper and 12 strips made from oilseed rape leaves were stuck on the periphery. The strips were about 38 mm wide and protruded about 30mm from the periphery like the teeth of a gear. Subsequently, the toothed disc was positioned in the laser beam in such a manner that while turning the laser beam alternatively targeted the leaf stripes (reflection level A) and the gaps in a radius of 200 mm. A shell filled with wet sand soil was positioned vertically and perpendicular to the beam to generate the reflection level B. For estimating the ratio from teeth (R_teeth_) of oilseed rape leaves to gaps (R_gaps_) a specific measurement with a triangulation sensor was performed. The movement of the acrylic glass disc was performed by a small electric drive. The circumferential speed steps were V_1_ = 1.67 m s^-1^ V_2_ = 3.34 m s^-1^, and V_3_= 6.70 m s^-1^ and therefore within the interval of ground speeds of agricultural machines.

### 2.5. Measurements under same conditions in a real crop

To test the measurement properties under real field conditions the laser rangefinder was mounted on a tool carrier together with a swivel drive. In the case of large measuring distances and swivel angles, it is very difficult to investigate the functional relationship between the reflection distance and crop biomass density. An exact site reference to the scanned area can be found only at a high expense. Laser rangefinders can be assessed however in terms of their measuring properties for greater distances by always scanning the same crop plants. Under field conditions, those requirements can be created by a laser sensor mounted on a base vehicle in such a way that its swivel axis is oriented perpendicular to the base vehicle. If the base vehicle is parked in a crop field, for each scan exactly the same crop biomass can be ensured. In this case, the laser sensor should measure the same mean reflection distance for each scan. Based on the variance of the mean reflection distance of each scan it is possible to assess the repeatability of measurements under constant crop conditions.

**Figure 4.**
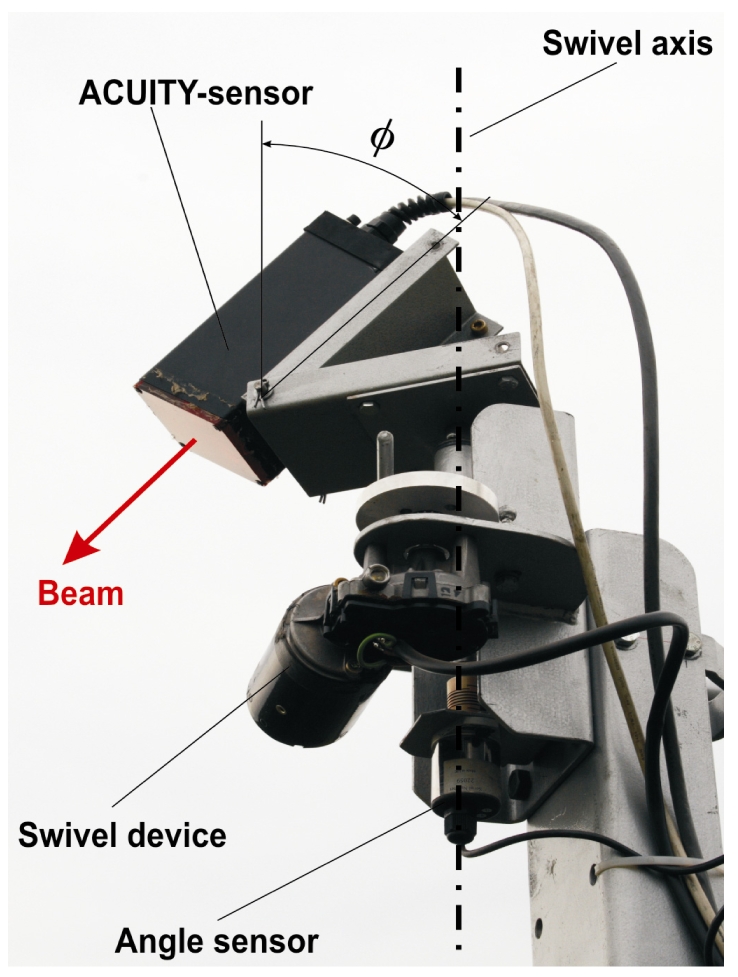
Arrangement of laser rangefinder and swivel drive (vertical axis) for measurements under field conditions.

Another way to assess the sensor properties for larger measuring distance, is to scan characteristics of the crop field as tram lines or stock edges. This should be marked by leaps in reflection distance in the individual scans clearly according to the corresponding swivel angles γ. The sensor was moved with a special swivel body (Figure 4). The swivel axis was arranged perpendicular to the base vehicle and it was swung with a crank arm on a swivel angle of 73 ° and with a frequency of about 1 Hz. The time-synchronous measurement of the swivel angle was performed with a P500A.160 L300 rotation angle sensor (Positek Ltd., UK). As inclination angles φ of the sensor, 45 °, 60 ° and 75 ° were chosen. The crop plots scanned were ripe winter wheat in the summer of 2007 and shooting winter wheat in the Spring of 2008.

## 3. Results and Discussion

### 3.1. Static accuracy

Growing and established crop plants have, in contrast to trees, a height in the range of decimetres. Therefore an accuracy at the millimetre level is necessary to measure crop parameters for agricultural production processes. The distance measurements for unmovable target areas like a white sheet of paper, an oilseed rape leaf and sand soil resulted in standard deviations for random errors in the range of millimetres (Table 3). A slight increase was observed for far distances. For most agricultural applications this measuring accuracy is sufficient because roll, pitch and yaw movements of the basic agricultural vehicle cause higher inaccuracies. The exact definition of a tolerable error limit cannot be given with the current state of knowledge.

### 3.2. Distribution of light intensity inside the beam cross section

**Figure 5.**
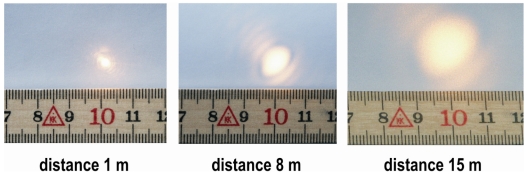
Distribution of light intensity inside the laser beam for three measuring distances.

According to the manufacturers' information the diameter of the laser spot (D_spot_) can be calculated approximately: D_spot_ ≈ 2.5 mm + 0.0005×(measuring range in mm). That means e.g. the spot has a theoretical diameter of about 7.5 mm in the range of 10.00 m. The measurements (Figure 5) confirmed the magnitude of beam cross section given by the manufacturer. Figure 5 demonstrates however that the distribution of light intensity differs from the concentric shape and therefore from the ideal Gauss beam cross section distribution. From this irregular distribution it results that the laser rangefinder has specific beam parameters and therefore specific measuring features. As a consequence of this the theoretical considerations on measuring properties will suffer.

### 3.3. Multiple reflection levels inside of the beam

**Figure 6.**
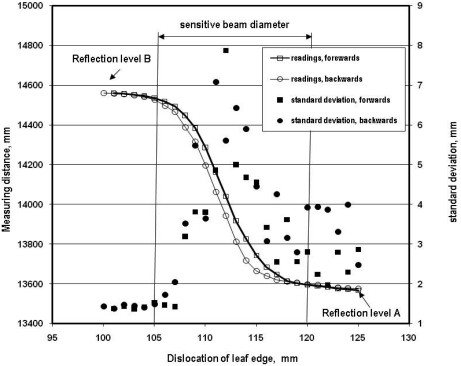
Distance readings of the laser rangefinder depending on leaf edge dislocation according to Figure 2.

Based on the measuring arrangement in Figure 2 increased standard deviations were observed when the sensitive laser beam targeted two reflection areas (oil seed rape leaf and soil surface) simultaneously. Figure 6 demonstrates this measuring property in the upper measurement range. When the beam targets either the leaf of oilseed rape or the soil surface, the standard deviation was within the range of the readings under static conditions. However, when the beam targets both reflection objects, the standard deviation increased up to 8 mm. Furthermore, from Figure 6 it can be concluded that the sensor readings reflect the relation of illuminated areas resulting from both reflection levels adequately. The influence of movement direction (forwards, backwards) of the leaf on measured distance and standard deviation was marginal. Serious outliers were observed, particularly if the reflection level A was close and reflection level B was further away from the sensor.

### 3.4. Dynamic measurements

For the assessment of measuring properties under dynamic conditions it was necessary to estimate in a first step the ratio from the oilseed rape leaves to the gaps between the leaves (Figure 3). This resulted in R_teeth_ = 0.365 to R_gaps_ = 0.635. From this the theoretical resulting measuring distance l_cal_ was calculated according to eqn. (1):

(1) $${\text{l}}_{\text{cal}}={\text{R}}_{\text{teeth}}l_{\text{A}}+{\text{R}}_{\text{gaps}}l_{\text{B}}$$

where l_A_ represents the distance from sensor to reflection level A (teeth from oilseed rape), in m, and l_B_ the distance from the sensor to reflection level B (soil surface), expressed in m (the distances between the sensor and levels A and B were estimated by the rangefinder readings themselves under static conditions.)

Table 4 shows the absolute and relative deviations from the calculated reference distances. The absolute deviations were in the centimetre range and the relative deviations were less than 1 %. Mostly the laser rangefinder measured a slightly higher distance then that calculated according to Eq. (1). An influence of the circumferential speed (equivalent to the vehicle ground speed) in the investigated interval was not recognisable. This can be explained mainly by the ratio for the speed of light (about 3x10^8^ ms^-1^) to the speed of target areas in the range of a few ms^-1^ and the co-axial arrangement of the transmitter and receiver of the sensor.

### 3.5. Tests under field conditions

**Figure 7.**
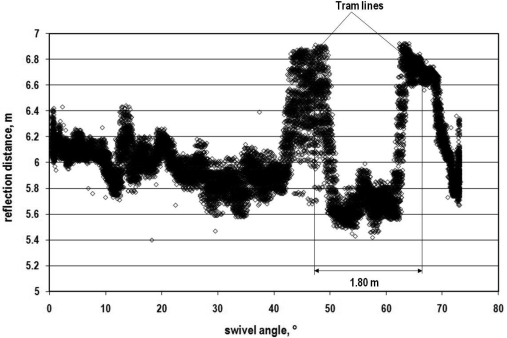
Example for the repeatability of 60 scans of the laser rangefinder in a real crop field (ripe winter wheat, inclination angle φ = 60°).

To demonstrate exemplarily the conformity of laser readings from the individual scans in visual form, the measured reflection distances and the corresponding swivel angles were combined (Figure 7). The crop was ripe winter wheat immediately before harvest. As Figure 7 expresses, the characteristic patterns of each scan were reflected. Particularly striking are the jumps, caused by the tram lines. In this figure, single points can be observed which obviously cannot result from the crop. Nevertheless, based on the high number of measuring points for each scan, the mean values of scans differ slightly. To assess the scans more quantitatively, the readings for both scanning directions were compared. This comparison, summarized in Table 5, shows that the mean reflection distance of the individual scans has a standard deviation in the range of centimetres for ripe winter wheat and millimetres for green winter wheat. The corresponding coefficients of variation were less than 1 %. An explanation for the reduced standard deviation in green winter wheat results from the used wavelength of 780 nm. At this wavelength green plants reflect the light very intensely.

In agriculture the exploitation of laser rangefinders for optimisation of production processes is just beginning. Resulting from the current state of knowledge laser rangefinders can be used successfully in a site specific technique for application of fertiliser and crop protection agents and also on harvesting machines. In the market-available agricultural machinery sector low cost laser rangefinders are installed on combine harvesters. For example, the agricultural engineering company CLAAS in Bielefeld (Germany) attaches a laser rangefinder (Laser pilot) on Lexion combines harvesters for detection of crop edges. Based on this information the steering mechanism keeps the optimum cutting width constant. A similar solution can be found on the newest CX-combine harvester from the CASE-NEW HOLLAND company (Lake Forest, IL, USA) named Smart-Steer. Besides the optimisation of cutting width the accuracy of yield mapping can be improved.

The laser rangefinders used on combine harvesters are not able to detect detailed crop parameters like, e.g., the crop biomass density. As shown in the introduction, the laser scanner systems used in the field of remote sensing are very cost and labour intensive and therefore not suitable for agricultural practices. In agricultural engineering research only a low cost laser scanner and laser rangefinder with a fixed beam (e.g. those of the SICK AG, company, Waldkirch, Germany and Schmitt Measurement Systems, Inc., USA) were investigated. As discussed in this paper, each sensor system has specific measurement features and generates inconsistent readings, so the comparison of readings gathered under different conditions is very limited.

For both remote sensing with expensive laser scanner systems and low cost laser scanners for agricultural applications it is necessary to discriminate between soil surface and plant material. Because of a lot of modelling experience in remote sensing science the developed methods should be tested and transferred to agriculture for crop assessment.

## 4. Conclusions

Under static conditions the investigated laser rangefinder measured distances with a standard deviation in the millimetre range. For measuring crop plants under field conditions this accuracy is quite sufficient. Compared to other influencing parameters like vibrations and movements of the basic vehicle this error source is of no importance. The influence of vehicle movements on measuring accuracy was not the object of this paper and should be quantified in further investigations. The increased cross section of the beam caused by longer ranges results in reduced measuring quality. Resulting from the increased footprint, gaps in the crop arrangement will not be clearly detected and the probability of multiple reflection levels will increase. Furthermore, the investigations demonstrated that multiple reflection levels generated reduced accuracy and even measurement failures. The measurements under field conditions in real crops demonstrated that in spite of the discussed sources of errors a high level of repeatability was achieved. This repeatability for higher measuring ranges of more than 2.5 m indicates that crops can be scanned successfully with a low cost laser rangefinder measuring only one echo. To reduce the probability of multiple echoes and to support the penetration of crop structure, the cross section of the beam should be minimised. To reduce the inhibition threshold for agricultural applications the price for such a sensor should be less than a few thousand Euros.

A second way to introduce suitable laser rangefinders in agriculture is the tremendous reduction of prices for multiple echo sensor systems, an increase of their robustness and minimisation of their size. The development of such sensors in the car industry for mass production could be a solution for agriculture in the future.

Further investigations are necessary to establish the potential of laser rangefinders in agriculture. Main areas of research should be the current analysis of progress in the laser rangefinder technology and sophisticated tests in scanning and modelling the most important agricultural crops under the aspect of improving the agricultural production process.

## Figures and Tables

**Table 1.** Coefficients of determination for the relationship between crop biomass density and mean reflection height for small ranges < 2.50 m [2]

**Crop cultivar**	**Growth stages**	**No. of plots**	**Regression**	**R^2^**
Oilseed rape	51-61	8	^1)^ h_Rmean_ = 0.090 FMD	0.92
		8	2) h_Rmean_ = 0.077 FMD	0.97
Winter rye	31-69	13	^1)^ h_Rmean_ = 0.146 FMD	0.91
		13	2) h_Rmean_ = 0.116 FMD	0.90
Winter wheat	30-59	10	^1)^ h_Rmean_ = 0.091 FMD	0.94
		10	2) h_Rmean_ = 0.074 FMD	0.96
Grassland	-	8	^1)^ h_Rmean_ = 0.153 FMD	0.61
		8	2) h_Rmean_ = 0.099 FMD	0.48

1) LASE-sensor

2) ACUITY-sensor

**Table 2.** Technical data of the sensor ACUITY AccuRange 4000-LIR (manufacturer's information).

Measuring range up to	16.50 m	Divergence	0.5 mrad
Wave length	780 nm	Laser output	20 mW
Measuring frequency	50,000 Hz	Classification	3B
Voltage internal	5 V	Length/ height /width	160/80/80 mm
Power requirement	1.5 W	Mass	0.624 kg
Laser spot size	2.5 mm	Price	€ 6,900
Linearity	2.5 mm		

**Table 3.** Standard deviations (STDW) and coefficients of variation (CV) for immobile target areas.

Measuring range m		**White sheet of paper**		**Leaf of oilseed rape**		**Sandy soil**	

		STDW mm	CV %	STDW mm	CV %	STDW mm	CV %
Short	1.00	0.53	0.053	0.48	0.048	0.82	0.082
Medium	8.00	0.53	0.007	1.27	0.016	1.09	0.014
Far	14.90	1.08	0.007	2.19	0.015	1.64	0.011

**Table 4.** Absolute and relative deviations of range readings from calculated value for two reflection levels and three circumferential speeds (see Figure 3).

**Range**	**Parameter**	**Unit**	**v_1_ = 1.67 ms^-1^**	**v_2_ = 3.34 ms^-1^**	**v_3_ = 6.70 ms^-1^**
Short	l_A_	m	1.500	1.500	1.500
	l_B_	m	2.000	2.000	2.000
	l_cal_	m	1.805	1.818	1.818
	l_m_	m	1.813	1.800	1.843
	l_cal_-l_m_	m	-0.008	0.018	-0.025
	100 (l_cal_-l_m_) l_cal_^-1^	%	-0.46	1.00	-1.38

Medium 1	l_A_	m	8.000	8.000	8.000
	l_B_	m	8.600	8.600	8.600
	l_cal_	m	8.364	8.381	8.381
	l_m_	m	8.368	8.414	8.426
	l_cal_-l_m_	m	-0.004	-0.033	-0.045
	100 (l_cal_-l_m_) l_cal_^-1^	%	-0.47	-0.40	-0.53

Medium 2	l_A_	m	8.000	8.000	8.000
	l_B_	m	11.000	11.000	11.000
	l_cal_	m	9.860	9.905	9.905
	l_m_	m	9.917	9.908	9.947
	l_cal_-l_m_	m	-0.060	-0.003	-0.041
	100 (l_cal_-l_m_) l_cal_^-1^	%	-0.57	-0.03	-0.42

Long	l_A_	m	13.500	13.500	13.500
	l_B_	m	14.500	14.500	14.500
	l_cal_	m	14.130	14.152	14.152
	l_m_	m	14.170	14.173	14.194
	l_cal_-l_m_	m	-0.041	-0.021	-0.041
	100 (l_cal_-l_m_) l_cal_^-1^	%	-0.29	-0.15	-0.29

l_m_ distance measured by the laser rangefinder

**Table 5.** Comparison of mean values, standard deviations, and coefficients of variation of reflection distance of scans in ripe and green winter wheat (forward / reverse motion).

**Inclination angle φ grad**	**number of scans**	**mean value m**	**STDW m**	**CV %**
winter wheat, ripe 13.07.2007, sensor height 3.65 m				
45	43 / 43	4.543 /4.564	0.0095 / 0.0084	0.21 / 0.18
60	60 / 60	6.061 / 6.065	0.0118 / 0.0116	0.19 / 0.19
75	56 / 56	10.093 /10.087	0.0538 / 0.0512	0.53 / 0.51
winter wheat, BBCH 33, 15.5.2008, sensor height 2.75 m				
45	24 / 22	3.214 / 3.188	0.0094 / 0.0086	0.29 / 0.16
60	25 / 23	4.064 / 4.080	0.0062 / 0.0086	0.15 / 0.21
75	26 / 25	7.213 / 7.127	0.0067 / 0.0087	0.09 / 0.12
